# Reducing the Mysteries of Sulfur Metabolism

**DOI:** 10.1371/journal.pbio.0030284

**Published:** 2005-07-19

Sulfur is one of life's essential atoms. It is found in two amino acids—methionine and cysteine—and in several vitamins. Outside of living organisms, sulfur is mostly found in its fully oxidized form, the sulfate ion (SO_4_^2−^). To incorporate it into biomolecules, enzymes must reduce it, stripping it of oxygens. As is the case with so many other metabolic pathways, microorganisms possess a richer and more varied sulfur metabolism than humans do. In particular, bacteria, including the human pathogen Mycobacterium tuberculosis, use enzymes called sulfonucleotide reductases to begin the reduction process, creating reduced sulfite (SO_3_^2−^). In this issue of *PLoS Biology*, Carolyn Bertozzi and colleagues elucidate the novel molecular mechanism at the heart of this reaction, and show that it is conserved among a wide variety of organisms, from bacteria to plants.

The process begins when sulfate links up with an ATP molecule to form a species, abbreviated APS. The authors used a variety of biochemical techniques to discover what happens next. When APS was incubated with one kind of sulfonucleotide reductase, called APS reductase, they found that the weight of the enzyme increased by 80 Daltons, exactly the weight of a covalently bound sulfite, suggesting that the sulfur of the APS had been reduced and linked to the enzyme. By mutating the enzyme, they showed that the sulfite links to a critical cysteine amino acid, which itself contains a sulfur (sulfur–sulfur covalent bonds are common in biochemical molecules). To regenerate the enzyme and liberate the sulfite, yet another sulfur-containing compound, thioredoxin, joins the action, the result of which is that sulfite is released and the enzyme is restored to its original form, ready to react again. [Fig pbio-0030284-g001]

**Figure pbio-0030284-g001:**
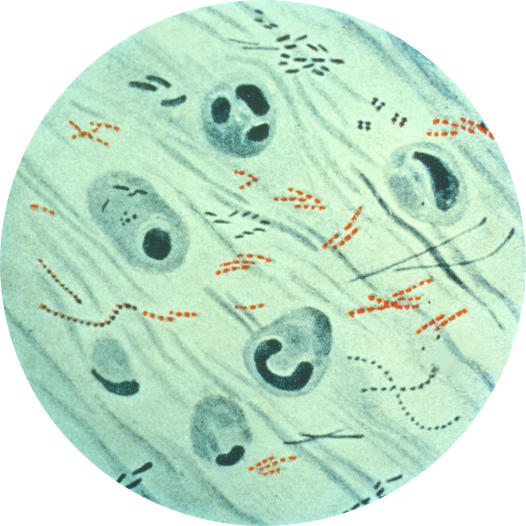
Researchers deduced the mechanism of the highly conserved sulfonucleotide reductase enzyme in Mycobacterium tuberculosis pictured here (Image: US Centers for Disease Control and Prevention)

Analysis of the same pathway in two other types of bacteria confirmed that this same mechanism occurs in each. Combined with previous observations of a similar mechanism in the model plant Arabidopsis, these results demonstrate a remarkable bit of functional conservation over many millions of years of evolution. As well as elucidating an important piece of sulfur biochemistry, the deeper understanding of this critical biochemical process provided by this study may have practical implications. Since humans do not possess this pathway, drugs that target it might make an effective antibiotic with a low risk for side effects.

